# The Role of Guideline’s Threshold Vascular Diameters in Long-Term Radio-Cephalic Arteriovenous Fistula Failure

**DOI:** 10.3390/jcm14134667

**Published:** 2025-07-01

**Authors:** Eliza Russu, Elena Florea, Alexandra Asztalos, Constantin Claudiu Ciucanu, Eliza-Mihaela Arbănași, Réka Bartus, Adrian Vasile Mureșan, Alexandru-Andrei Ujlaki-Nagy, Ioan Hosu, Emil-Marian Arbănași

**Affiliations:** 1 Department of Vascular Surgery, George Emil Palade University of Medicine, Pharmacy, Science and Technology of Targu Mures, 540139 Targu Mures, Romania; eliza.russu@umfst.ro (E.R.); claudiu.ciucanu@umfst.ro (C.C.C.); reka.kaller@umfst.ro (R.B.); adrian.muresan@umfst.ro (A.V.M.); emil.arbanasi@umfst.ro (E.-M.A.); 2 Clinic of Vascular Surgery, Mures County Emergency Hospital, 540136 Targu Mures, Romania; dr.florea.elena@gmail.com; 3 First Infectious Disease Clinic, Mureș County Clinical Hospital, 540139 Targu Mures, Romania; 4 Doctoral School of Medicine and Pharmacy, George Emil Palade University of Medicine, Pharmacy, Science and Technology of Targu Mures, 540139 Targu Mures, Romania; arbanasi.eliza@gmail.com; 5 Regenerative Medicine Laboratory, Centre for Advanced Medical and Pharmaceutical Research (CCAMF), George Emil Palade University of Medicine, Pharmacy, Science and Technology of Targu Mures, 540139 Targu Mures, Romania; 6 Psychiatry Clinic No. 2, Mureș County Clinical Hospital, 540139 Targu Mures, Romania; u.alex8@yahoo.com; 7 Department of Nephrology, Mureș County Emergency Hospital, 540136 Targu Mures, Romania; ioan.hosu@umfst.ro

**Keywords:** arteriovenous fistula, ESVS, vascular surgery, RC-AVF, vascular mapping, dialysis, vascular access

## Abstract

**Background/Objectives**: According to the guidelines established by the European Society of Vascular Surgery (ESVS), a minimum 2 mm diameter is advised for both the radial artery (RA) and cephalic vein (CV) to perform a radio-cephalic arteriovenous fistula (RC-AVF). However, studies have suggested that larger vein diameters, over 2.5 or 3 mm, or even smaller vessel diameters, above 1.6 mm, can yield satisfactory outcomes in both the medium and long term. This study aims to analyze how preoperative vascular mapping influences the long-term RC-AVF failure, considering adherence to guidelines. **Methods**: This retrospective, monocentric, and observational study enrolled 110 patients with ESKD who were admitted, between 2018 and 2024, to the Vascular Surgery Department at the Emergency County Hospital of Târgu Mureș for the creation of an RC-AVF. Demographic characteristics, comorbidities, preoperative vascular mapping data, and laboratory data were meticulously collected from the hospital’s electronic databases. Patients enrolled in the current study were categorized into two groups based on their adherence to guideline recommendations. **Results**: Patients whose RC-AVF was created outside guideline recommendations demonstrated smaller arterial (*p* < 0.001) and venous (*p* < 0.001) diameters. Additionally, a higher percentage of these patients were on hemodialysis via CVC at the time of RC-AVF creation (*p* = 0.041), as well as a higher incidence of 6-week AVF maturation failure (*p* = 0.012) and long-term AVF failure (*p* = 0.016). In ROC Curve analysis, a threshold of 2.75 mm was established for the RA (AUC: 0.647, *p* = 0.005) and 2.52 mm for the CV (AUC: 0.677, *p* = 0.001). Additionally, patients whose RC-AVF procedures adhered to guideline recommendations had a significantly lower risk of long-term RC-AVF failure (HR: 0.44, *p* = 0.012). This association lost significance after adjusting for cardiovascular risk factors and the presence of CVC at admission (HR: 0.69, *p* = 0.328). After full adjustment, only the CV remained an independent predictor of long-term successful RC-AVF (HR: 0.68, *p* = 0.026). In contrast, RA lost significance after adjusting for cardiovascular risk factors and the presence of CVC at admission (HR: 0.71, *p* = 0.086). **Conclusions**: In conclusion, this study reveals that only the diameter of the CV is correlated with the long-term failure of RC-AVF, independent of age, gender, diabetes, hypertension, active smoking, and the presence of a CVC at the time of AVF creation. Therefore, while adhering to the threshold diameters of the AR and CV, as recommended by the ESVS guidelines, facilitates the creation of a functional RC-AVF, we assert that additional cofactors, such as demographic data, usual cardiovascular risk factors, or CVC presence, must also be considered to achieve optimal long-term AVF.

## 1. Introduction

According to the World Health Organization (WHO), chronic kidney disease (CKD) is a global health problem that has a negative impact on health systems and the quality of life of patients [1], especially in less developed countries [2]. In 2016, it was estimated that approximately 850 million people suffered from this disease, and it is expected to become the fifth leading cause of death globally by 2040 [3]. Unfortunately, patients with end-stage chronic kidney disease (ESKD) depend on renal replacement therapy (RRT) for survival, which may involve hemodialysis (HD) or peritoneal dialysis (PD) [4]. However, patients with ESKD undergoing long-term hemodialysis require optimal vascular access. Among the available vascular access (VA) options, the preferred choice is an arteriovenous fistula (AVF) due to its superior primary patency and lower postoperative complication rate compared to the other two options: arteriovenous graft (AVG) and central venous catheter (CVC) [5,6,7,8,9].

According to the guidelines established by the European Society of Vascular Surgery (ESVS), it is advised to perform a radio-cephalic arteriovenous fistula (RC-AVF) at the level of the non-dominant limb, as a primary intention, 3 to 6 months prior to the anticipated commencement of hemodialysis [5]. Despite advancements in surgical techniques and the devices utilized in creating the RC-AVF, its long-term performance remains suboptimal [5]. Consequently, as indicated by existing literature and reported in the ESVS guideline, the RC-AVF is associated with an early failure rate ranging from 5% to 46%, with a one-year secondary patency rate varying between 42% and 83% [10,11,12,13,14,15,16,17,18,19]. In the articles referenced in the guidelines, different thresholds for cephalic vein (CV) diameter, such as 1.6 mm [17], 2.5 mm [10,14], or 3 mm [12,18], have been utilized in the establishment of RC-AVF. In contrast, most studies [10,14,17] have generally accepted a threshold of 2 mm for the radial artery (RA). Although the ESVS guideline recommends a minimum 2 mm diameter for both RA and CV for the surgical creation of RC-AVF, some studies have suggested that larger [10,12,14,16] or even smaller [18,20,21] vessel diameters can yield satisfactory outcomes in both the medium and long term.

This study aims to analyze how preoperative vascular mapping influences the long-term RC-AVF failure, considering adherence to guidelines. Furthermore, we will evaluate the risk factors and ascertain the optimal thresholds for RA and CV in our cohort associated with AVF failure.

## 2. Materials and Methods

### 2.1. Study Design

This study is a retrospective, monocentric, observational analysis of all patients with ESKD who were admitted, between 2018 and 2024, to the Vascular Surgery Department at the Emergency County Hospital of Târgu Mureș for the creation of an RC-AVF. Patients lacking complete information in the hospital’s electronic database, as well as those without recorded preoperative vessel diameters and those who underwent proximal RC-AVF, were excluded from this study. Additionally, patients whose postoperative RC-AVF showed no thrill, as well as those who did not initiate dialysis using the newly created RC-AVF, were also excluded. Patients who underwent intentional surgical closure of the RC-AVF for various reasons, those who were lost to follow-up, and patients who died during that period were also excluded. Patients enrolled in the current study were categorized into two groups based on their adherence to guideline recommendations.

### 2.2. Data Collection

Demographic characteristics, comorbidities, and laboratory data were meticulously collected from the hospital’s electronic databases. The following comorbidities were taken into consideration: hypertension, atrial fibrillation (AF), ischemic heart disease (IHD), history of myocardial infarction, diabetes, history of stroke, and active smoking. In terms of laboratory data, the following parameters were evaluated prior to surgery: hemoglobin, hematocrit, potassium, sodium, creatinine, blood urea nitrogen (BUN), neutrophil count, lymphocyte count, monocyte count, platelet count, and glucose level.

### 2.3. Preoperative Vascular Mapping

Preoperatively, each patient underwent ultrasound evaluation to ascertain the location of the RC-AVF. The linear probe of the ultrasound machine (12–5 MHz, Samsung HS60; Seoul, Republic of Korea) facilitated the assessment of vessels regarding their quality and diameter from the wrist joint to the cubital area. Additionally, after designating and marking the area for the creation of the RC-AVF, the diameters of the CV and RA were measured at that level without the application of a tourniquet.

### 2.4. RC-AVF Creation

All fistulas were performed by the same surgical team under local anesthesia through a small incision proximal to the wrist. Following meticulous dissection, the radial artery and cephalic vein were identified and clamped. In all cases, the anastomosis was conducted end-to-side (vein-to-artery) utilizing continuous suturing with 6-0 sutures. After confirming the presence of the trill through the vein, the subcutaneous and dermal tissues were closed in layers. Moreover, the surgical wound was covered with sterile adhesive bandages.

### 2.5. Follow-Up

The primary endpoint of the study is the failure of the RC-AVF that occurs following dialysis initiation at the level of the newly created AVF, which is defined as the inability to conduct dialysis sessions. To monitor the progress of patients, we meticulously reviewed their medical records and directly contacted the chronic dialysis centers. Comprehensive information regarding all patients was gathered until the occurrence of AVF failure or until 31 January 2025. Following the creation of the RC-AVF, patients were followed up for a mean period of 1.98 ± 1.45 years, with a maximum follow-up period of 5.06 years.

### 2.6. Statistical Analysis

SPSS for Mac OS version 29.0.2.0 was used for statistical analysis (SPSS, Inc., Chicago, IL, USA). Age, follow-up period, and arterial and venous diameter are reported as mean values with standard deviation (SD). Additionally, all laboratory data are reported as the median and quartile 1 (Q1)—quartile 3 (Q3). To evaluate the differences in continuous variables, we utilized the Mann–Whitney and Student’s *t*-tests. The Chi-square test was performed to examine the differences between the dichotomous variables. Furthermore, we applied Receiver Operating Characteristic (ROC) curve analysis to explore the relationship between preoperative vascular diameter and AVF failure. Kaplan–Meier survival curves were used to analyze the relationship between adherence to guideline recommendations and RC-AVF failure, as well as the association between the optimal cut-off values of the RA and CV with respect to RC-AVF failure. The log-rank test was used to compare the curves. Cox-regression analyses were used to evaluate the predictive role of variables of interest in relation to RC-AVF failure. Furthermore, we employed four distinct adjustment models to evaluate the relationships among RC-AVF established in accordance with guideline recommendations, artery and vein diameters, and long-term AVF failure. Specifically, Model 1 is unadjusted; Model 2 incorporates age and sex; Model 3 incorporates age, sex, and cardiovascular risk factors (including diabetes, hypertension, and active smoking); and Model 4 additionally considers the presence of CVC at the time of RC-AVF creation. All tests were two-tailed, and a *p*-value less than 0.05 was considered statistically significant.

## 3. Results

In the current study, 110 patients with RC-AVF were enrolled, with a mean age of 62.70 ± 14.46 years, of whom 62 patients (56.36%) were male, while 48 patients (43.64%) were female. Within the entire cohort, 87 patients (79.09%) had their RC-AVF constructed following guideline recommendations, whereas 23 patients (20.91%) did not adhere to these recommendations. Among the most prevalent cardiovascular comorbidities, we identified hypertension in 99 patients (90.0%) and ischemic heart disease in 50 patients (45.45%). Additionally, 44 patients (40.0%) were diabetic, and 18 patients (16.36%) were current smokers. Concerning AVF creation, it was performed in the nondominant upper limb for 94 patients (85.45%), while 65 patients (59.09%) had their AVF created in outpatient settings. At admission, 51 patients (46.36%) were undergoing hemodialysis via a CVC (Table 1). Preoperatively, vascular mapping indicated a mean diameter of the radial artery of 2.71 ± 0.75 mm and a mean diameter of the cephalic vein of 2.81 ± 0.61 mm. During the follow-up period, 6 weeks postoperation, 23 patients (20.91%) failed to meet maturation criteria, and in the long term, 43 patients (39.09%) experienced AVF failure (Table 1).

No demographic, comorbidity, or preoperative laboratory data differences were found between the two groups of patients. Patients whose RC-AVF was created outside guideline recommendations demonstrated smaller arterial (*p* < 0.001) and venous (*p* < 0.001) diameters during preoperative vascular mapping, as well as smaller 6-week arterial diameters (*p* = 0.003). Additionally, a higher percentage of these patients were on hemodialysis via CVC at the time of RC-AVF creation (*p* = 0.041). During the follow-up, there was a higher incidence of 6-week AVF maturation failure (*p* = 0.012) and long-term AVF failure (*p* = 0.016) in the same group (Table 1).

**Figure 1:**
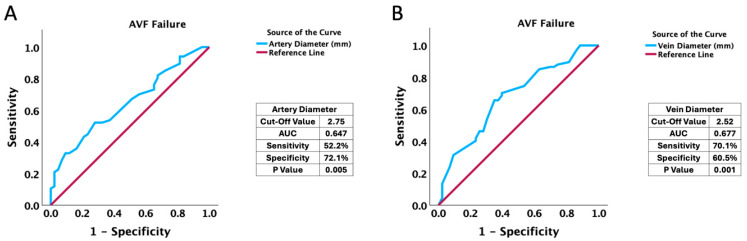
The characteristics of ROC curve analysis in relation to long-term AVF failure concerning (**A**) artery diameter and (**B**) vein diameter.

Furthermore, during the ROC curve analysis, we identified the optimal cut-off values for the radial artery and cephalic vein in relation to long-term RC-AVF failure within our cohort. As illustrated in Figure 1, we determined thresholds of 2.75 mm for the radial artery (AUC: 0.647, *p* = 0.005) and 2.52 mm for the cephalic vein (AUC: 0.677, *p* = 0.001).

Table 2 presents the sensitivity, specificity, and Youden’s index for various diameters of the radial artery and cephalic vein, as determined by ROC curve analysis. Accordingly, based on established guidelines, a diameter of at least 2 mm for the radial artery in the current study demonstrates an 85.1% sensitivity but only a 27.9% specificity. Similarly, sensitivities of 89.6% and specificities of 18.6% were observed for the cephalic vein.

**Figure 2:**
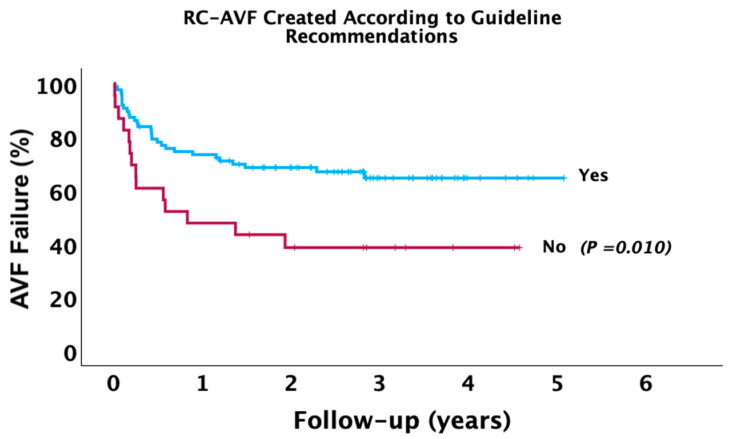
Kaplan–Meier survival curve for the incidence of RC-AVF failure during follow-up in the entire cohort based on AVF creation in alignment with guideline recommendations.

**Figure 3:**
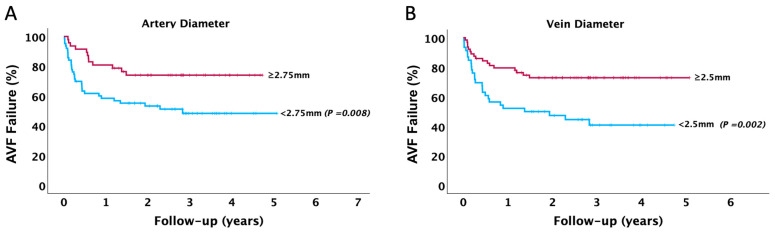
Kaplan–Meier survival curve for the incidence of RC-AVF failure during follow-up in the entire cohort based on the optimal cut-off value of (**A**) artery diameter and (**B**) vein diameter.

At Kaplan–Meier, patients with RC-AVF created following guideline recommendations demonstrated lower long-term AVF failure (log-rank *p* = 0.010) (Figure 2). Furthermore, patients with preoperative arterial (log-rank *p* = 0.008) and venous (log-rank *p* = 0.002) diameters exceeding the optimal threshold identified in this study demonstrated lower long-term AVF failure (Figure 3).

Cox regression analysis indicated that females (HR: 2.12, *p* = 0.015), diabetes (HR: 1.96, *p* = 0.027), active smokers (HR: 2.84, *p* = 0.002), and the presence of CVC at the time of admission (HR: 2.49, *p* = 0.004) were significantly associated with long-term failure of the RC-AVF (Table 3).

Additionally, patients whose RC-AVF procedures adhered to guideline recommendations had a significantly lower risk of long-term RC-AVF failure (HR: 0.44, *p* = 0.012), which supports the clinical utility of existing vascular access guidelines. This association remained valid even after adjusting for demographic factors such as age and sex (HR: 0.51, *p* = 0.043), but it lost significance when accounting for cardiovascular risk factors and the presence of CVC at admission (HR: 0.69, *p* = 0.328) (Table 4). Moreover, a higher artery diameter measured during preoperative vascular mapping was associated with lower long-term RC-AVF failure (HR: 0.56, *p* = 0.005) in the unadjusted model and maintained a significant association after adjusting for age and sex (HR: 0.60, *p* = 0.013). However, this significance disappeared when adjusted for age, sex, cardiovascular risk factors, and CVC presence (HR: 0.71, *p* = 0.086) (Table 4). In contrast to artery diameter, vein diameter maintained a consistent and statistically significant association with reduced RC-AVF failure across all models: unadjusted (HR: 0.61, *p* = 0.002) and fully adjusted, including demographics, cardiovascular risk factors, and CVC presence (HR: 0.68, *p* = 0.026) (Table 4).

## 4. Discussion

The primary findings of the current study highlight that adhering to established guidelines when performing RC-AVF procedures ensures the creation of a functional vascular access for dialysis. Although the vessel’s diameter is an important factor, it should not be the sole criterion for surgical decision-making, as cardiovascular comorbidities, risk factors, and CVC presence have a negative impact on the long-term performance of RC-AVF. It also highlights that vascular mapping, particularly venous diameter, plays an important role in predicting long-term outcomes. According to our study, a minimum threshold diameter of 2.52 mm for CV is linked to a reduced long-term failure rate of RC-AVF, as established by the fully adjusted model in multivariate analysis. Additionally, in univariate analysis, variables such as female gender, diabetes, active smoking, and the presence of CVC at the time of VA creation are correlated with long-term failure of RC-AVF. Therefore, a comprehensive, individualized approach to patient selection and surgical planning, considering both anatomical and clinical factors, is essential for improving the success rates of RC-AVF.

While the ESVS guidelines primarily recommend RC-AVF as the first AVF option, it presents a higher risk of maturation failure and long-term dysfunction compared to more proximal AVF [5,10,11,12,13,14,15,16,17,18,19]. Various innovations, surgical techniques, and devices have been proposed and evaluated to improve the long-term performance of RC-AVF [22]. Initially, various techniques were examined, including the suture line method, anastomotic approach, anastomotic angle, and vessel preparation type [22]. In their study, Aitken et al. [23] conducted a randomized controlled trial (RCT) comparing interrupted and continuous suturing techniques in patients with RC-AVF. They found no significant improvement in functional patency (52% vs. 36%, *p* = 0.18). For vessel anastomosis, two approaches were used: end-to-side (ES) and side-to-side (SS). A recent RCT involving 100 patients who underwent various types of AVF revealed that the ES technique led to better functional maturation (*p* = 0.0001), but there was no notable difference in 1-year secondary patency (*p* = 0.225) [24]. In the past decade, two devices have been proposed to enhance haemodynamic flow: Optiflow [25] and VasQ [26]. However, only VasQ has undergone analysis in an RCT, revealing a higher functional patency associated with the device (*p* = 0.01), as well as a lower incidence of stenosis (*p* = 0.04) [26]. Therefore, limited innovations have significantly influenced the long-term performance of RC-AVF. In the current study, all patients have benefited from ES anastomosis, employing continuous suture and slit arteriotomy, performed by the same surgical team.

The key factors for achieving optimal RC-AVF involve the quality and diameter of the RA and CV [27,28,29,30,31]. It is widely recognized that diabetes negatively affects the RA, leading to significant atherosclerotic deposits that impede arterial flow and subsequently influence the performance of RC-AVF [31]. The threshold diameter of RA proposed by the ESVS guideline is 2 mm [5], which was recently validated in a meta-analysis published by Kordzadeh et al. [32]. In contrast, the current study identified an optimal cut-off value of 2.75 mm for RA (52.2% sensitivity and 72.1% specificity). However, in the Cox regression analysis, the association between RA diameter and long-term AVF failure was no longer significant after adjustment for cardiovascular risk factors.

According to the current study, the CV has a more important impact than RA on the patency of RC-AVF. As shown in Table 4, the preoperative vein diameter is associated with long-term AVF failure, independent of demographic data, cardiovascular risk factors, and the presence of a CVC at the time of RC-AVF creation (HR: 0.68, *p* = 0.026). Regarding the quality of the CV wall, Kaller et al. [33] observed that intimal hyperplasia (IH) and angiogenesis are associated with RC-AVF maturation failure, analyzing intraoperative CV specimens from 42 patients. Furthermore, the same authors identified, through ROC curve analysis, a threshold of 2.25 mm for RA and 2.55 mm for CV regarding the maturation failure [29]. Furthermore, Park et al. [34] found that a minimum CV diameter of 2 mm correlated with primary maturation (OR: 9.572, *p* = 0.006) and primary patency (HR: 0.273, *p* = 0.004) of RC-AVF in non-diabetic patients, although the significance for primary patency (HR: 0.561, *p* = 0.078) was not confirmed across all patients. Likewise, Hou et al. [35] observed in multivariate analysis that a CV diameter exceeding 2 mm predicted successful RC-AVF (HR: 4.55, *p* = 0.008). However, the authors found no link between RA diameter and AVF functionality.

In a large cohort of 277 Asian patients, Li et al. [36] determined optimal cutoff diameters for CV of 1.85 mm and for RA of 2.05 mm. However, it is essential to note that in the Asian patient population, hemodialysis is typically administered at a flow rate of 250 mL/min [37]. Before the dissemination of the vascular access ESVS guidelines, the Dutch Vascular Access Study Group conducted a multicentric study across eight hospitals, analyzing the risk factors associated with RC-AVF maturation failure in a sample of 1383 patients [38]. The authors observed in their multivariate logistic regression analysis that a CV diameter of less than 2.5 mm (OR: 1.53, *p* = 0.044) and female gender (OR: 2.20, *p* < 0.001) were associated with arteriovenous fistula (AVF) maturation failure [38]. Consistent with previous findings, the current study confirmed that female gender (HR: 2.12, *p* = 0.015) was associated with long-term AVF maturation failure over the long term. Furthermore, as indicated by the Kaplan–Meier survival curve, patients with a CV diameter of less than 2.5 mm exhibit an increased risk of long-term primary patency failure (log-rank *p* = 0.002). In a recent study, Heindel et al. [39] reported that female sex (HR: 1.21, *p* = 0.034), diabetes (HR: 1.21, *p* = 0.039), pre-hemodialysis (HR: 0.69, *p* < 0.001), and vein diameter of less than 3 mm (HR: 1.33, *p* = 0.013) were linked to an earlier and more frequent need for interventions. It is essential to note that, while the AUC values for RA (0.647) and CV (0.677) suggest a limited capacity for association with AVF failure, a threshold value for CV equal to or exceeding that identified in the current study has been validated in several studies [10,14,38].

While the findings of this study are significant, it is essential to acknowledge its limitations, which should be taken into account when interpreting the results. Firstly, this research is a retrospective, observational, single-center study with a limited patient sample. Moving forward, we suggest conducting prospective multicenter studies to validate these findings. Secondly, due to the retrospective nature of our study, we were unable to find information in the hospital’s electronic database regarding other postoperative ultrasound results or chronic medications. Consequently, we were unable to incorporate data related to AVF flow or the severity of IH into our analysis, as well as the influence of antidiabetic, statin, or other chronic medication usage on the patency of RC-AVF into our analysis. Another significant limitation arises from the lack of information regarding postoperative complications and the mid- and long-term causes of AVF failure, including cannulation issues, infections, pseudoaneurysms, AVF stenosis, and AVF thrombosis resulting from hypotension or cardiac problems. Finally, the information on the hemodynamic evaluation of RC-AVF at six weeks was not available for the analysis because nephrologists conducted the maturation assessment according to our hospital’s protocol.

## 5. Conclusions

In conclusion, the present study demonstrates that vessel diameters, as determined through preoperative vascular mapping, are correlated with improved long-term performance of RC-AVF. While adherence to the threshold diameters of the RA and CV as recommended by the ESVS guideline facilitates the creation of a functional RC-AVF, it should not serve as the sole criterion for clinical decision-making, in our opinion. Furthermore, our research suggests that cardiovascular comorbidities, risk factors, and the presence of CVC have a negative impact on the long-term performance of RC-AVF. Additionally, this study reveals that only the diameter of the CV is correlated with the long-term failure of RC-AVF, independent of age, gender, diabetes, hypertension, active smoking, and the presence of a CVC at the time of AVF creation. Therefore, a comprehensive, individualized approach to patient selection and surgical planning, considering both anatomical and clinical factors, is essential for improving the success rates of RC-AVF.

## Figures and Tables

**Table 1:** All characteristics of patients enrolled in this study presented for the entire cohort and according to the creation of RC-AVF according to the guideline recommendation.

Variables	All Patients n = 110	RC-AVF Created in Agreement with Guideline Recommendation		*p* Value
		Yes n = 87	No n = 23	
Age, mean ± SD	62.70 ± 14.46	63.39 ± 13.49	60.13 ± 17.77	0.419
Male, no. (%)	62 (56.36%)	52 (59.77%)	10 (43.48%)	0.161
Female, no. (%)	48 (43.64%)	35 (40.23%)	13 (56.52%)	
Comorbidities and risk factors, no. (%)				
Hypertension	99 (90.00%)	78 (89.66%)	21 (91.30%)	0.815
Atrial Fibrillation	7 (6.36%)	4 (4.60%)	3 (13.04%)	0.140
Diabetes	44 (40.00%)	33 (37.93%)	11 (47.83%)	0.389
Ischemic Heart Disease	50 (45.45%)	37 (42.53%)	13 (56.52%)	0.231
History of Myocardial Infarction	5 (4.55%)	5 (5.75%)	0 (0.00%)	0.236
History of Stroke	4 (3.64%)	2 (2.30%)	2 (8.70%)	0.145
Active Smoking	18 (16.36%)	12 (13.79%)	6 (26.09%)	0.156
Laboratory data, median (Q1–Q3)				
WBC	7.90 (6.36–9.50)	8.04 (6.50–9.54)	7.01 (6.11–9.30)	0.969
Potassium mmol/l	5.05 (4.63–5.44)	5.01 (4.58–5.43)	5.10 (4.69–5.47)	0.380
Sodium mmol/l	139 (137–141)	139 (137.07–141)	139.45 (137–140)	0.239
Glucose (mg/dL)	102 (89.42–131.22)	101 (89–128.9)	105 (96–174)	0.080
BUN (mg/dL)	122.40 (99.30–160.20)	120.60 (92–157.30)	137.40 (102.42–174.3)	0.890
Creatinine (mg/dL)	6.32 (5.26–7.81)	6.10 (5.25–7.29)	6.71 (5.66–8.27)	0.240
Hemoglobin g/dL	10.00 (8.7–11.4)	10.00 (8.7–11.41)	10.35 (8.63–11)	0.313
Hematocrit %	30.50 (26.37–35.1)	30.40 (26.82–35.02)	30.89 (25.92–35.27)	0.287
Neutrophils ×10^3^/uL	5.40 (4.16–7.11)	5.49 (4.33–7.31)	4.80 (3.71–6.47)	0.295
Lymphocytes ×10^3^/uL	1.51 (1.06–2.07)	1.44 (1.01–2.07)	1.72 (1.18–1.96)	0.555
Monocyte ×10^3^/uL PLT ×10^3^/uL	0.64 (0.52–0.80) 220.50 (181–296.07)	0.63 (0.51–0.81) 213.5 (177.77–282.5)	0.66 (0.56–0.79) 277 (191.25–305.75)	0.651 0.358
Vascular mapping determinations, mean ± SD				
Arterial Diameter (mm)	2.71 ± 0.75	2.87 ± 0.71	2.06 ± 0.48	**<0.001**
Vein Diameter (mm)	2.81 ± 0.61	2.96 ± 0.54	2.19 ± 0.46	**<0.001**
6-week Arterial Diameter (mm) ^#^	2.69 ± 0.73	2.85 ± 0.73	2.24 ± 0.52	**0.003**
6-week Vein Diameter (mm) ^#^	5.96 ± 1.51	6.05 ± 1.43	5.71 ± 1.75	0.249
Non-Dominant Upper Limb	94 (85.45%)	74 (85.06%)	20 (86.96%)	0.818
Out-Patients, no. (%)	65 (59.09%)	50 (57.47%)	15 (65.22%)	0.502
CVC present, no. (%)	51 (46.36%)	36 (41.38%)	15 (65.22%)	**0.041**
6-week Maturation failure, no. (%)	23 (20.91%)	14 (16.09%)	9 (39.13%)	**0.012**
Long-Term AVF failure	43 (39.09%)	29 (33.33%)	14 (60.87%)	**0.016**
Follow-up Period (Years), mean ± SD	1.98 ± 1.45	2.10 ± 1.40	1.53 ± 1.55	0.083

^#^ the values of the 6-week vein and artery diameters are available only for a group of 41 patients from the entire cohort; the *p*-values highlighted in bold indicate statistically significant differences (*p* < 0.05).

**Table 2:** Coordinates of radial artery and cephalic vein diameter for the area under the curve (AUC).

Radial Artery				Cephalic Vein			
Diameter	Sensitivity	Specificity	Youden’s Index	Diameter	Sensitivity	Specificity	Youden’s Index
2.05 mm	85.1%	27.9%	0.130	2.05 mm	89.6%	18.6%	0.082
2.37 mm	68.7%	46.5%	0.152	2.35 mm	85.1%	37.2%	0.223
2.55 mm	53.7%	62.8%	0.160	2.45 mm	74.6%	46.5%	0.211
2.65 mm	52.2%	67.4%	0.207	**2.52 mm**	**70.1%**	**60.5%**	**0.306**
**2.75 mm**	**52.2%**	**72.1%**	**0.243**	2.57 mm	68.7%	60.5%	0.291
2.85 mm	44.8%	76.7%	0.215	2.65 mm	65.7%	62.8%	0.285
2.95 mm	43.3%	79.1%	0.224	2.75 mm	65.7%	65.1%	0.304
3.05 mm	35.8%	83.7%	0.205	2.90 mm	53.7%	69.8%	0.235
3.13 mm	32.8%	88.4%	0.212	3.08 mm	46.3%	74.4%	0.207
3.18 mm	32.8%	90.7%	0.235	3.25 mm	34.3%	86.0%	0.204
3.25 mm	28.4%	93.0%	0.214	3.35 mm	31.3%	90.7%	0.220

The Values highlighted in bold indicate the optimal cut-off value.

**Table 3:** Cox regression analysis of the risk factors and long-term RC-AVF failure.

Variables	RC-AVF Failure		
	HR	95% CI	*p* Value
Female	2.12	1.15–3.90	**0.015**
Hypertension	0.45	0.20–1.01	0.053
Ischemic Heart Disease	1.24	0.76–2.01	0.380
Diabetes	1.96	1.08–3.59	**0.027**
Active Smoking	2.84	1.48–5.48	**0.002**
CVC presence	2.49	1.33–4.67	**0.004**
6-week Maturation Failure	3.31	1.76–6.23	**<0.001**

The *p*-values highlighted in bold indicate statistically significant (*p* < 0.05).

**Table 4:** Multivariate analysis of preoperative vascular mapping characteristics and long-term RC-AVF failure.

Variables		RC-AVF Failure		
		HR	95% CI	*p* Value
RC-AVF Created in Agreement with Guideline Recommendation (Yes)	Model 1	0.44	0.23–0.83	**0.012**
	Model 2	0.51	0.26–0.98	**0.043**
	Model 3	0.59	0.29–1.19	0.146
	Model 4	0.69	0.34–1.43	0.328
Artery Diameter	Model 1	0.56 *	0.38–0.84	**0.005**
	Model 2	0.60 *	0.41–0.89	**0.013**
	Model 3	0.68 *	0.46–1.01	0.059
	Model 4	0.71 *	0.48–1.05	0.086
Vein Diameter	Model 1	0.61 *	0.44–0.84	**0.002**
	Model 2	0.62 *	0.45–0.85	**0.004**
	Model 3	0.67 *	0.48–0.94	**0.022**
	Model 4	0.68 *	0.49–0.96	**0.026**

* HR expressed per 1 SD increase in baseline artery and vein diameter. The *p*-values highlighted in bold indicate statistical significance (*p* < 0.05). Model 1: unadjusted. Model 2: age and sex. Model 3: age, sex, and CV risk factors (diabetes, hypertension, active smoking). Model 4: age, sex, CV risk factors (diabetes, hypertension, active smoking), and CVC presence.

## Data Availability

The data that support the findings of this study are available from the corresponding author upon reasonable request.

## Abbreviations

The following abbreviations are used in this manuscript:

ESVS	European Society of Vascular Surgery
RA	radial artery
CV	cephalic vein
RC-AVF	radio-cephalic arteriovenous fistula
WHO	World Health Organization
CKD	chronic kidney disease
ESKD	end-stage chronic kidney disease
RRT	renal replacement therapy
HD	hemodialysis
PD	peritoneal dialysis
VA	vascular access
AVF	arteriovenous fistula
AVG	arteriovenous graft
CVC	central venous catheter
AF	atrial fibrillation
IHD	ischemic heart disease
BUN	blood urea nitrogen
SD	standard deviation
ROC	Receiver Operating Characteristic
RCT	randomized controlled trial
